# Diversity, Host Specialization, and Geographic Structure of Filarial Nematodes Infecting Malagasy Bats

**DOI:** 10.1371/journal.pone.0145709

**Published:** 2016-01-11

**Authors:** Beza Ramasindrazana, Koussay Dellagi, Erwan Lagadec, Milijaona Randrianarivelojosia, Steven M. Goodman, Pablo Tortosa

**Affiliations:** 1 Centre de Recherche et de Veille sur les maladies émergentes dans l’Océan Indien, Plateforme de Recherche CYROI, Sainte Clotilde, La Réunion, France; 2 Université de La Réunion, UMR PIMIT "Processus Infectieux en Milieu Insulaire Tropical", INSERM U 1187, CNRS 9192, IRD 249. Plateforme de Recherche CYROI, 97490 Sainte Clotilde, Saint-Denis, La Réunion, France; 3 Association Vahatra, Antananarivo, Madagascar; 4 Institut Pasteur de Madagascar, Antananarivo, Madagascar; 5 The Field Museum of Natural History, Chicago, Illinois, United States of America; Northern Illinois University, UNITED STATES

## Abstract

We investigated filarial infection in Malagasy bats to gain insights into the diversity of these parasites and explore the factors shaping their distribution. Samples were obtained from 947 individual bats collected from 52 sites on Madagascar and representing 31 of the 44 species currently recognized on the island. Samples were screened for the presence of micro- and macro-parasites through both molecular and morphological approaches. Phylogenetic analyses showed that filarial diversity in Malagasy bats formed three main groups, the most common represented by *Litomosa* spp. infecting *Miniopterus* spp. (Miniopteridae); a second group infecting *Pipistrellus* cf. *hesperidus* (Vespertilionidae) embedded within the *Litomosoides* cluster, which is recognized herein for the first time from Madagascar; and a third group composed of lineages with no clear genetic relationship to both previously described filarial nematodes and found in *M*. *griveaudi*, *Myotis goudoti*, *Neoromicia matroka* (Vespertilionidae), *Otomops madagascariensis* (Molossidae), and *Paratriaenops furculus* (Hipposideridae). We further analyzed the infection rates and distribution pattern of *Litomosa* spp., which was the most diverse and prevalent filarial taxon in our sample. Filarial infection was disproportionally more common in males than females in *Miniopterus* spp., which might be explained by some aspect of roosting behavior of these cave-dwelling bats. We also found marked geographic structure in the three *Litomosa* clades, mainly linked to bioclimatic conditions rather than host-parasite associations. While this study demonstrates distinct patterns of filarial nematode infection in Malagasy bats and highlights potential drivers of associated geographic distributions, future work should focus on their alpha taxonomy and characterize arthropod vectors.

## Introduction

Knowledge of Malagasy bats has improved considerably during the last two decades, with 44 bat species currently recognized on the island, of which about 75% are endemic [1–3]. In addition, a number of studies have focused on the ecology, biology, and biogeography of these animals, providing substantial insights into their evolutionary biology and natural history [4–10]. Certain research programs with Malagasy bats have increasingly integrated other fields of study such as parasitology, microbiology, and virology. These multidisciplinary investigations aim to understand the role of bats as reservoirs of microorganisms of possible medical importance [11–13] and explore drivers of host-parasite associations [14, 15].

Research undertaken in different parts of the world to explore metazoan endoparasites of bats has revealed an important diversity of helminthes [16–18], including nematodes [18, 19]. This latter group is of particular interest, as its high diversity allows investigations addressing the evolutionary history and medical importance of these animals. Indeed, nematodes represent the second-most diversified animal group on our planet after arthropods [20], with over 25,000 described species, including about 1,200 infecting vertebrates [20–23], some specifically bats [16, 24, 25] and terrestrial small mammals [19]. Nematode infection rarely results in the host’s death; however, infection may affect the physiology and behavior of parasitized animals [20]. Additionally, experimental studies carried out with filarial nematodes parasitizing animals provide insights into the biology and developmental characteristics of the most common human filariasis, such as *Wuchereria bancrofti* (Cobbold, 1877), *Brugia malayi* S. L. Brug, 1927, or *Onchocerca volvulus* Bickel, 1982 [20, 26].

In bats, 34 genera of nematodes have been reported [20], among which *Litomosa* and *Litomosoides* (Onchocercidae) are the best known, at least based on morphology [24, 27–29]. The genus *Litomosa* infects different bat families including the Hipposideridae, Miniopteridae, Molossidae, Pteropodidae, Rhinolophidae, and Vespertilionidae [28–31]. In South Africa, for example, *L*. *chiropterorum* Ortlepp, 1932 was redescribed from *Miniopterus natalensis* populations (Miniopteridae) based on morphology and molecular genetics [24].

On Madagascar, the investigation of bat blood parasites led to the morphological identification of three main groups: microfilaria, haemoproteids, and trypanosomes [32]. Subsequently, a new species of filaria, *L*. *goodmani* Martin, Bain, Jouvenet, Raharimanga, Robert & Rousset, 2006, was described based on morphology [30]. The holotype of this nematode was recovered from *M*. *gleni* collected in northern Madagascar, Parc National d’Ankarana, Andrafiabe Cave (12°55’S, 49°03’E). These authors also reported from the same locality a female filarial specimen closely related to *L*. *goodmani* and recovered from *M*. “*manavi*”; based on subsequent taxonomic revisions, the host is now referable to *M*. *aelleni* [33]. While morphological differences were observed, the taxonomic identity of this female adult filaria was not addressed and the species was reported as *Litomosa* sp.

Studies overlying morphological and molecular tools on parasitic nematodes have yet to be conducted on Malagasy bats and information on filarial diversity, phylogeny, and distribution are poorly known. Given the high levels of endemism of the island’s bat fauna, including for most groups a good understanding of their colonization history, geographic distributions, and speciation patterns [1, 5, 34], sufficient information is now available to examine the drivers of these host-parasite associations. Herein, we explore filarial infection in Malagasy bats using PCR detection, phylogenetic analysis, and, to a lesser extent, morphological characterization. Following molecular identification of filarial taxa, we investigate the role of different variables including bioclimate, roosting ecology, and geographic distribution of infected bat species in the occurrence of filarial parasites.

## Materials and Methods

### Ethic Statement

The procedures performed in this study were not subjected to the approval of an ethics committee or to specific national or international regulations at the time of sampling. This study was conducted in strict accordance with the terms of research permits issued by Malagasy authorities (Direction du Système des Aires Protégées, Direction Générale de l’Environnement et des Forêts and Madagascar National Parks; permits numbers 194/12/MEF/SG/DGF/DCB.SAP/SCB, 067/12/MEF/SG/DGF/DCB.SAP/SCBSE and 032/12/MEF/SG/DGF/DCB.SAP/SCBSE) and following national laws. Animals were captured, manipulated, and euthanized with thoracic compression following guidelines accepted by the scientific community for the handling of wild mammals [35]. The only exception was *Pteropus rufus*, individuals of which were injected with a euthanizing agent. With the exception of *P*. *rufus*, the samples collected in the wild did not include any species covered by international treaties, such as CITES. For *P*. *rufus*, a CITES Appendix II species, specimens were purchased alive in a market and were not physically collected by the research team in a natural setting. A CITES permit from the Malagasy national authority was issued for tissue export (permit 243C-EA06/MG12) to the CRVOI laboratory on La Réunion.

### Bat sampling

**Fig 1:**
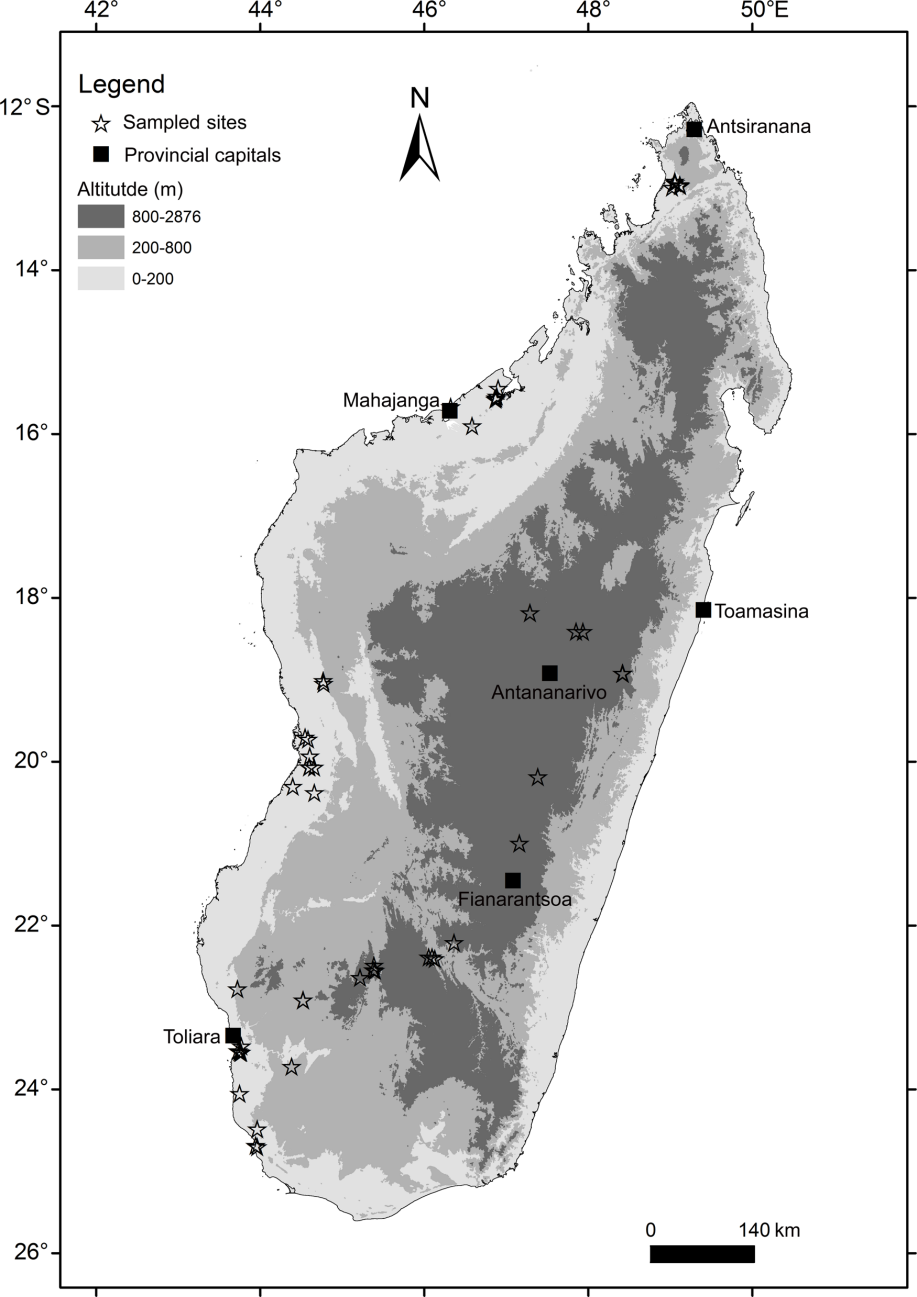
Localization of the different sampling sites on Madagascar overlaid on elevation.

In total, 52 sites across Madagascar were visited between February 2012 and March 2013, with a strong bias to the western and central portions of the island. This geographic bias is in part associated with the island’s geology and the roosting ecology of many bat species, as there are no significant sedimentary formations in the east and in the few shallow caves of this region, bat density and diversity are notably low compared to the limestone and sandstone areas of the west [36]. Bats were captured using mist nets and harp traps installed at cave entrances and across foraging pathways, as well as direct collection from a range of natural and synanthropic day-roost sites (Fig 1, S1 Table). This sampling of Malagasy bats is part of a large multidisciplinary research program aiming to advance studies of bat ecology and taxonomy [37], as well as ectoparasite diversity and evolution [38] and host bacterial and viral pathogens [15].

Upon capture, individual bats were placed in separate clean cloth bags and provisionally identified using morphological criteria. Information on external measurements, sex, reproductive status, and microhabitat were recorded. Voucher specimens were deposited at the Université d’Antananarivo, Département de Biologie Animale (UADBA), Antananarivo, Madagascar and at the Field Museum of Natural History (FMNH), Chicago, USA. Tissue samples from individual bats for pathogen research were placed in cryogenic tubes, frozen in liquid nitrogen, and then transported to the laboratory, where they were stored at –80°C.

### Adult filarial sampling and microscopic analyses

Adult filaria were directly recovered from each bat host during field dissection and subsequently stored in vials containing 90–95% ethanol. A thin blood smear was prepared from each bat specimen to document the morphological diversity of microfilaria circulating in the blood. After air-drying, blood smears were fixed with methanol for 10 s and stained with Giemsa solution before screening under an optical microscope at 100 and 400x magnification (Oxion, Euromex, Netherlands). Microscopic screening of blood smears was only conducted on animals displaying positive results from Polymerase Chain Reactions (PCRs, see below) and primarily to understand morphological variation of microfilaria.

### DNA Extraction and PCR amplification of bat filaria

A pool of approximately 1 mm^3^ of frozen kidney, lung, and spleen tissue from individual bat specimens was crushed in DMEM medium using two 3-mm tungsten beads in a TissueLyser II (Qiagen, Valencia, CA, USA) [13]. Subsequently, the mixture was centrifuged at 10,000 rpm for 5 min and the supernatant used for DNA extraction. Genomic DNA was extracted using an EZ1 robot with the viral mini kit v2.0 according to manufacturer’s protocol (Qiagen, Valencia, CA, USA). A 648-bp fragment of the mitochondrial cytochrome C oxidase subunit I (COI) gene was PCR amplified with primers COIintF and COIintR as described elsewhere [22, 39, 40]. All PCRs were conducted in 25-μl reactions containing 12.5 μl of GoTaq Hot Start Green Master Mix (Promega, Madison, WI, USA), 1 μl of each primer, 1 μl of DNA template, and 9.5 μl of nuclease-free water. The amplification profile was 94°C for 5 min followed by 40 cycles of 45 s at 94°C, 45 s at 52° and 90 s at 72°C. Five μl of each PCR product was loaded on a 1.7% agarose gel stained with 1X GelRed dye (Biotium Inc., CA, USA) and visualized after electrophoresis under UV light. Amplicons were sequenced at Genoscreen (Lille, France) using both forward and reverse primers.

### Sequences and phylogenetic analyses

Resulting COI sequences together with those available on GenBank were automatically aligned using MAFFT implemented in *Geneious* 6.1.4 (Biomatters, available from http://www.geneious.com/). All new sequences were easily aligned as there were no apparent insertions or deletions. All COI sequences produced in this study were submitted to GenBank under accession numbers KP728027-KP728094 (S2 Table). The alignment is provided as a supplementary Nexus file (S1).

The best-fit model of nucleotide substitution was determined using jModelTest based on the smallest value of Akaike Information Criterion (AIC) [41, 42] and phylogenetic reconstruction was carried out using Bayesian inference with MrBayes 3.2.1 [43, 44]. This later analysis consisted of two independent runs of four incrementally reactions (three hot and one cold) and Metropolis-Coupled Markov Chain Monte Carlo (MC^3^) starting from a random tree. MC^3^ was allowed to run for 5,000,000 generations with trees and associated model parameters sampled every 500 generations. The initial 2500 trees were discarded as burn-in and the harmonic mean of the likelihood was calculated by combining the two independent runs.

### Statistical analyses

A Pearson Chi-square test was used to investigate differences in the infection rates between male and female bats. We tested the association between *Litomosa* and their bat hosts to determine potential host-parasite interactions and associations using Parafit as implemented in the APE package [45] under R software version 3.0.0 [46]. As only a single sequence per lineage can be used as input in Parafit, one consensus sequence was generated for each *Litomosa* clade. Since there have been major taxonomic revisions of Malagasy bats over the past decade, especially within the genus *Miniopterus*, we used only one recent cytochrome *b* sequence (775 bp) for each bat host species (downloaded from GenBank; see S3 Table). Phylogenies of bat hosts and *Litomosa* parasites were constructed using PhyML with 1000 replicates on Seaview version 4.4.1 [47] using to the best-fit substitution models proposed by jModelTest. These host-parasite associations were subsequently edited in TreeMap v3.0 software [48]. Finally, we tested the correlation between collecting locality (latitude, longitude) and genetic distances of the *Litomosa* clades using a Mantel test implemented in the ADE4 package [49] under R version 3.0.0 [46].

## Results

### Filarial nematode infection in Malagasy bats

In total, 947 samples representing at least 31 bat taxa belonging to six families (Emballonuridae, Hipposideridae, Miniopteridae, Molossidae, Pteropodidae, and Vespertilionidae) were screened for the presence of filarial nematodes. Molecular detection by end-point PCR revealed that 64 (6.8%) individuals were infected. Further, 47 individual miniopterid bats hosted adult stages of filarial nematodes based on visual inspection during specimen dissection. The combined molecular screening and adult nematodes samples revealed 83 (8.8%) positive individual bats (Table 1). Nematode infection was largely restricted to the genus *Miniopterus*, with males showing higher rates than females (X^2^ = 15.930, P < 0.001, d.f. = 1, Table 1). All eight species of *Miniopterus* tested were found positive for filaria, although infection rates were variable. In addition to *Miniopterus* spp., four species tested positive for infection by PCR–*Otomops madagascariensis* (Molossidae), *Myotis goudoti* and *Neoromicia matroka* (Vespertilionidae), and *Paratriaenops furculus* (Hipposideridae), but no adult nematode was recovered from any of these species. All Emballonuridae and Pteropodidae samples tested negative for nematodes (Table 1).

**Table 1:** Filarial nematodes infection rates in Malagasy bats.

Family	Species	Tested individuals (male/female)	PCR positive individuals (male/female)	Number of adult filaria	Total number of detected filaria	Infection rates per species	Total infection rates	Infection status
**Pteropodidae**	*Eidolon dupreanum*	6/5	0/0	0	0	0.0	0.0	Not infected
	*Pteropus rufus*	10/10	0/0	0	0	0.0	0.0	
	*Rousettus madagascariensis*	12/37	0/0	0	0	0.0	0.0	
**Hipposideridae**	*Hipposideros commersoni*	3/24	0/0	0	0	0.0	0.0	Not infected except *P*. *furculus*
	*Paratriaenops furculus*	5/9	1/0	0	1	7.1	0.1	
	*Triaenops menamena*	42	0/0	0	0	0.0	0.0	
**Emballonuridae**	*Coleura kibomalandy*	1/2	0/0	0	0	0.0	0.0	Not infected
	*Paremballonura tiavato*	2/4	0/0	0	0	0.0	0.0	
**Miniopteridae**	*Miniopterus aelleni*	4/3	0/2	1	2	28.6	0.2	Infected
	*Miniopterus gleni*	12/10	2/1	0	3	13.6	0.3	
	*Miniopterus griffithsi*	5/2	1/0	0	1	14.3	0.1	
	*Miniopterus griveaudi*	39/77	3/3	3	9	5.2	0.6	
	*Miniopterus mahafaliensis*	75/14	28/2	31	43	33.7	3.2	
	*Miniopterus majori*	4/3	4/2	6	6	85.7	0.6	
	*Miniopterus manavi*	19/0	5/0	2	6	26.3	0.5	
	*Miniopterus sororculus*	4/18	3/2	2	5	22.7	0.5	
**Molossidae**	*Chaerephon atsinanana*	20/14	0/0	0	0	0.0	0.0	Not infected except *O*. *madagascariensis*
	*Chaerephon leucogaster*	41/53	0/0	0	0	0.0	0.0	
	*Mops leucostigma*	39/28	0/0	0	0	0.0	0.0	
	*Mops midas*	9/10	0/0	0	0	0.0	0.0	
	*Mormopterus jugularis*	121/31	0/0	0	0	0.0	0.0	
	*Otomops madagascariensis*	15/24	0/1	1	2	2.6	0.1	
**Vespertilionidae**	*Hypsugo bemainty*	1/1	0/0	0	0	0.0	0.0	Rarely infected
	*Myotis goudoti*	22/26	0/1	0	1	2.1	0.1	
	*Neoromicia malagasyensis*	1/1	0/0	1	1	0.0	0.0	
	*Neoromicia matroka*	2/1	1/0	0	1	33.3	0.1	
	*Neoromicia robertsi*	2/0	0/0	0	0	0.0	0.0	
	*Pipistrellus hesperidus*	7/4	0/0	0	0	0.0	0.0	
	*Pipistrellus raceyi*	1/2	0/0	0	0	0.0	0.0	
	*Pipistrellus* cf. *hesperidus*	3/5	0/2	0	2	25.0	0.2	
	*Scotophilus marovaza*	1/0	0/0	0	0	0.0	0.0	
	**Total**	**504/443**	**48/16**	**47**	**83**	** **	**6.8**	

### Malagasy bats share a diversity of filarial nematodes

**Fig 2:**
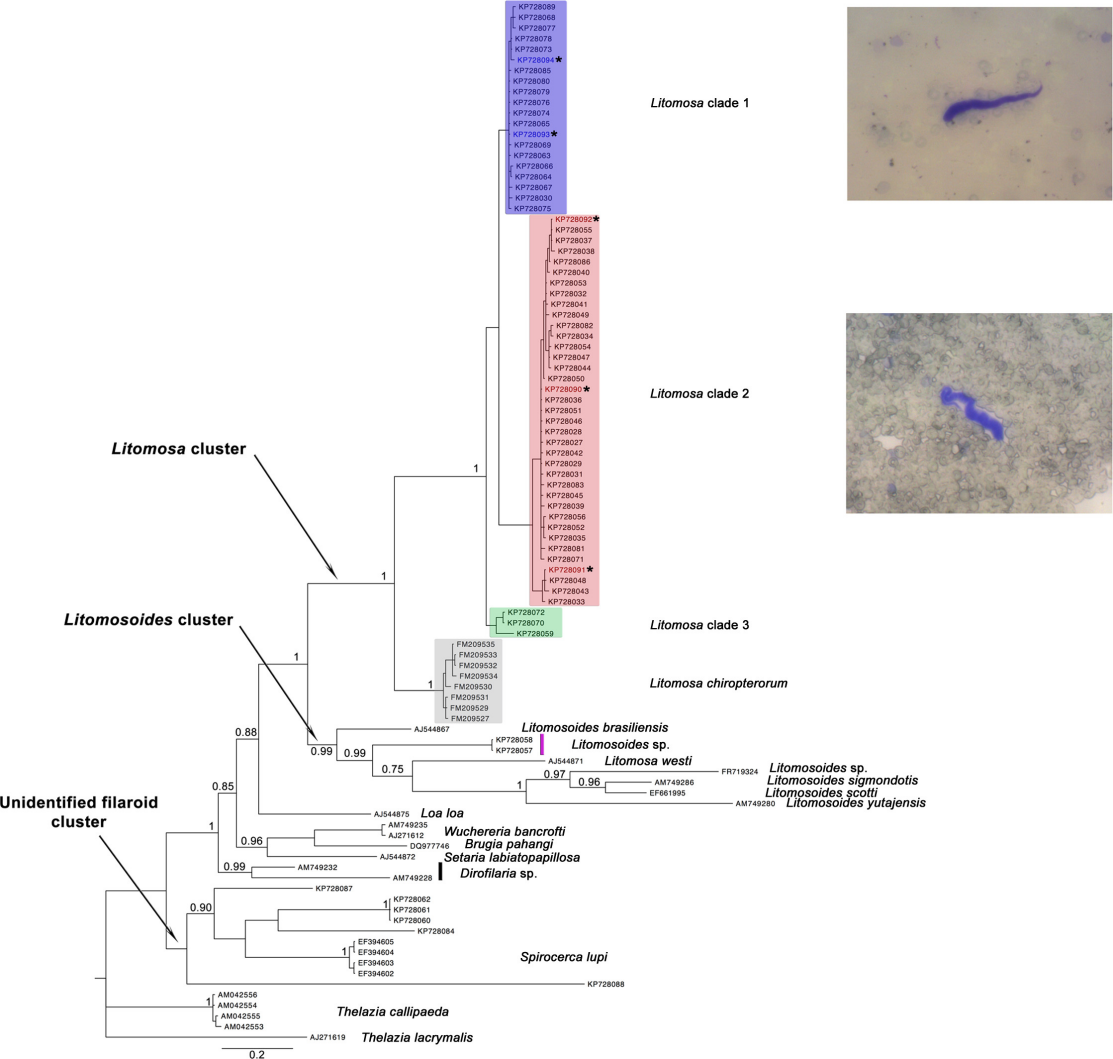
Bayesian phylogenetic tree based on mitochondrial COI sequences. Only posterior probabilities > 0.7 are presented. *Litomosa* lineages are outlined in color. Sequences obtained from adult filaria are indicated by an asterisk.

We performed a phylogenetic analysis to address the genetic diversity of nematodes infecting Malagasy bats and their relationships. For this analysis, we generated 63 COI sequences from positive samples and five sequences from adult filaria obtained from *Miniopterus mahafaliensis* and *M*. *manavi*. Further, we included 30 sequences downloaded from GenBank (see accession numbers in S2 Table). The sequence obtained from the single positive *Paratriaenops furculus* specimen was not included in the phylogenetic analysis, as it was very divergent from the other taxa presented in this study. Bayesian analysis using GTR+I+G as the best-fit substitution model revealed that filarial diversity in Malagasy bats segregated into three distinct groups referred to herein as *Litomosa* cluster, *Litomosoides* cluster, and “unidentified filaroid cluster”—this latter was quite diversified and included *Spirocerca lupi* (Fig 2).

The *Litomosa* cluster clearly represented the most diverse and prevalent filaria in Malagasy bats. This well-supported cluster (posterior probability, PP = 1) was composed of three Malagasy *Litomosa* lineages, referred to herein as clades 1, 2, and 3, obtained from all eight sampled *Miniopterus* spp., and of a sister species (*L*. *chiropterorum*) previously reported from South African *M*. *natalensis*. The separation of the South African *L*. *chiropterorum* from the three Malagasy *Litomosa* clades was well supported (PP = 1.00). *Litomosa* clade 3 was clearly separated from clades 1 and 2 (PP = 1.00), while the separation between clades 1 and 2 was not fully supported (PP = 0.66). From a host perspective, *Litomosa* clade 1 was obtained from *M*. *griveaudi*, *M*. *majori*, *M*. *manavi* sensu stricto, *M*. *gleni*, and *M*. *sororculus*; *Litomosa* clade 2 from *M*. *mahafaliensis*, *M*. *griffithsi*, and *M*. *sororculus*; and *Litomosa* clade 3 from *M*. *aelleni* and *M*. *griveaudi*. Hence, *M*. *griveaudi* was found infected with *Litomosa* belonging to all three clades; this species was also found infected with other undescribed filaria occurring within the “unidentified filaroid” cluster (see below, Fig 2). While microfilaria within *Litomosa* clades 1 and 2 were observed on thin blood smears (Fig 2), no microfilaria associated with *Litomosa* clade 3 was identified. Morphological studies on adult filaria from the three clades should help elucidate the taxonomy of *Litomosa* spp. infecting Malagasy bats, specifically those of the genus *Miniopterus*.

The *Litomosoides* cluster included two sequences obtained from female *Pipistrellus* cf. *hesperidus*. These two sequences are nested within the *Litomosoides* group, previously unknown from Madagascar (see below).

An “unidentified filaroid” cluster composed of sequences obtained from filaria infecting Malagasy bats, namely *Myotis goudoti*, *Miniopterus griveaudi*, *Neoromicia matroka*, and *Otomops madagascariensis*, also included the filaria *Spirocerca lupi* (Fig 2, S2 Table), which is known to infect carnivorans, notably canids and wild felids [50]. However, this clade was only marginally supported (PP = 0.68) and the absence of adults impedes further characterization.

### Bat filaria host-specificity

**Fig 3:**
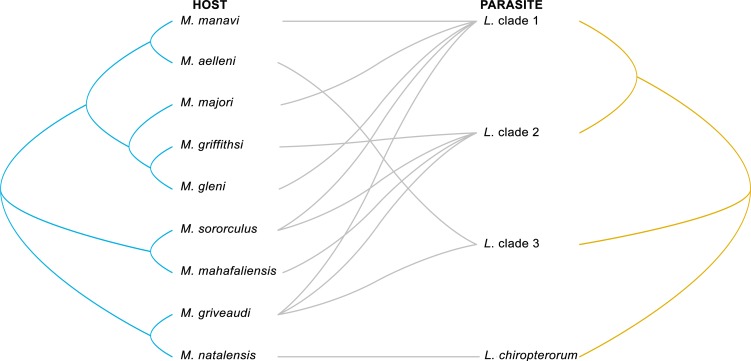
Host-parasite associations between *Miniopterus* spp. (Cyt *b*) and Afro-Malagasy *Litomosa* spp. (COI). Phylogenies were created using the HKY+G and HKY+I substitution model, respectively.

Our data show strong levels of specificity associated with the host genus, as *Litomosa* was found only in *Miniopterus* spp. and a *Litomosoides-*related lineage only infecting *Pipistrellus* cf. *hesperidus*. To further analyze relationships between host species and their nematode parasites, we used the *Miniopterus*-*Litomosa* associations from South Africa and Madagascar, as filaria of this genus were the most prevalent and genetically diverse within our dataset. We tested the null hypothesis of coevolution between filarial species (i.e. *Litomosa* clades 1, 2, 3, and *L*. *chiropterorum*) and their *Miniopterus* hosts by overlaying the two phylogenies. Parafit was used to test host-parasite coevolution of all 12 *Miniopterus*-*Litomosa* associations. The test revealed neither an overall significant host-parasite association (ParaFitGlobat = 0.005, P = 0.364 for 999 permutations, Fig 3) or any statistically significant association between a bat taxon and filarial parasite species (P > 0.05).

### Bioclimate and geographic structure of *Litomosa* infecting *Miniopterus* bats on Madagascar

We tested possible geographic structure of *Litomosa* genetic diversity using a Mantel test. This revealed a positive correlation between geographic distances separating sampling sites and genetic distances of the *Litomosa* obtained at these sites (Mantel test, *r* = 0.58, P < 0.001, 10000 repetitions). Hence, although the geographic distribution of *Litomosa* was found significantly structured across the island, the correlation coefficient was not sufficiently high to demonstrate a clear segregation of the different lineages based only on geography. In fact, there was some geographic overlap in *Litomosa*, with clade 3 always occurring in sympatry with either clade 1 or clade 2.

**Fig 4:**
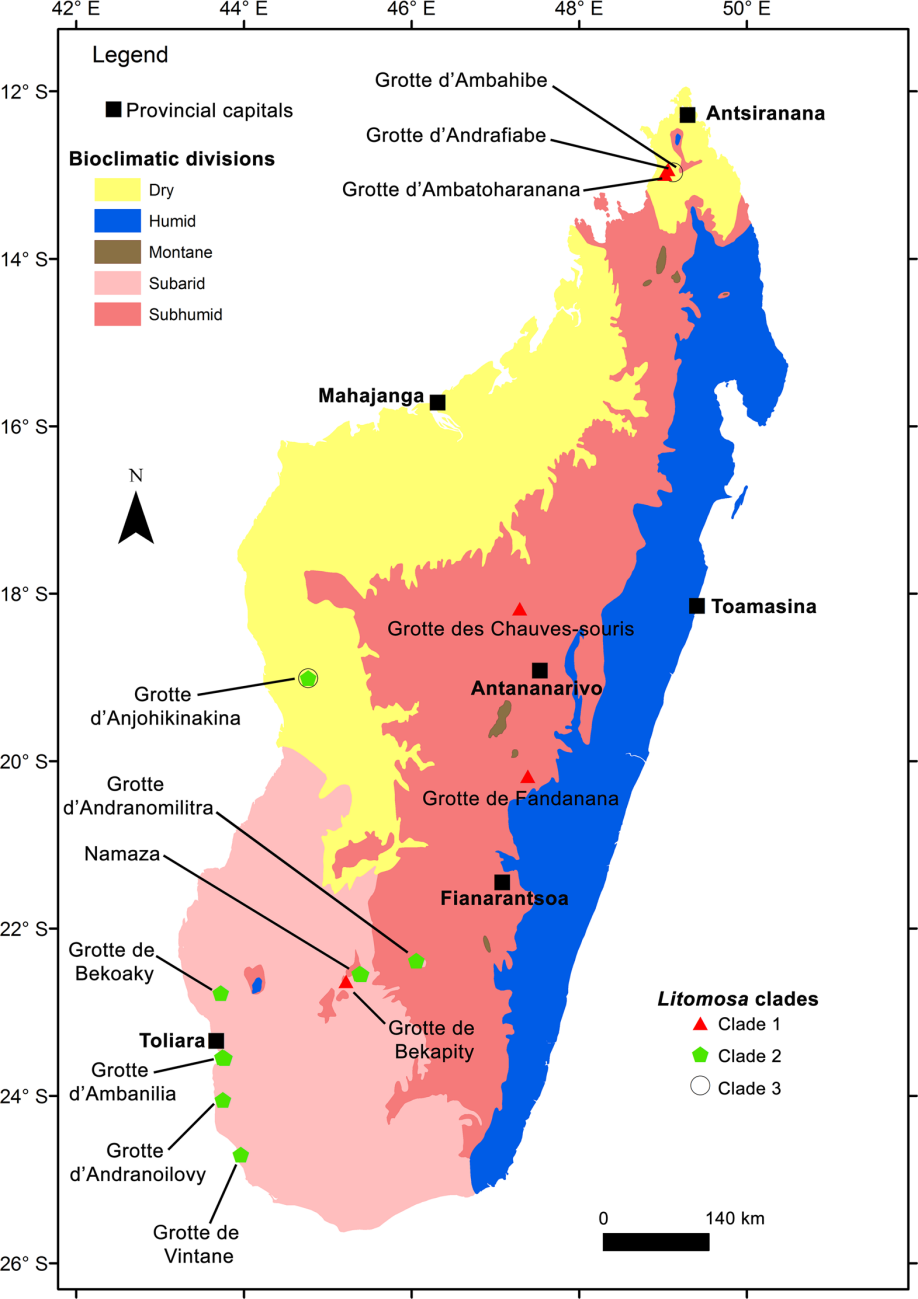
Geographic distribution of the three Malagasy *Litomosa* clades identified from *Miniopterus* spp. overlaid on bioclimatic regions of the island.

In Fig 4, we present the distribution of each *Litomosa* clade overlaid on the bioclimatic zones of Madagascar [51]. In general, clade 1 is prevalent in the Central and Northern Highlands (characterized by a subhumid climate), clade 2 is largely limited to the southwestern subarid and western dry areas, and clade 3 occurs in the dry climatic areas of the western and northwestern dry coastal areas (Table 2).

**Table 2:** Variation in the number of *Miniopterus* bats infected by the different *Litomosa* clades based on bioclimatic regions (see Fig 4).

*Litomosa* clades	Dry	Subarid	Subhumid	Total
**Clade 1**	4	0	14	18
**Clade 2**	1	33	0	34
**Clade 3**	3	0	0	3
**Negative**	122	72	37	231
**Total**	**130**	**105**	**51**	**286**

At a few sampling regions, two clades co-occur, specifically at Isalo (southwestern edge of the Central Highlands), Bemaraha (central west), and Ankarana (extreme north). At Isalo, the co-occurrence of two distinct parasites was observed in the same host species, *Miniopterus sororculus*, found infected with parasites belonging to either *Litomosa* clade 1 (from one individual at Bekapity) or *Litomosa* clade 2 (from several animals at Namaza). Noteworthy, co-occurrence of parasites belonging to both clades was not recorded in either of these two caves, which are separated by 19 km direct distance. In our sample, *Litomosa* sp. belonging to clade 3 was always sympatric with one of the two other *Litomosa* clades. For example, *M*. *griveaudi* from Bemaraha (Anjohikinakina) was found infected with both *Litomosa* clades 2 and 3, while *Litomosa* sp. belonging to clades 1 and 3 were recovered from *M*. *griveaudi* from two different caves in the Ankarana (Ambahibe and Andrafiabe, respectively) separated by 10 km direct distance (Fig 4, see S1 Table for GPS positions).

## Discussion

Recent studies on Malagasy bats using molecular and morphological characters have provided new data on the systematic relationships for most of the 44 bat species currently recognized on the island [1–3]. In the case of the genus *Miniopterus*, an explicit phylogeny has been published [52]. Information from extensive field inventories on the island together with the study of museum specimens has provided sufficient data for the development of species distribution models for the majority of recognized taxa [34]. These analyses have uncovered a number of biotic and abiotic variables that help explain with some precision the distribution of different taxa.

Over the past decade, a substantial amount of biological material has been collected from Malagasy bats to examine microorganisms—some of which are pathogenic—infecting these animals and provide insights into the role bats may play in their maintenance [12–15]. The present study provides an overview of filarial nematodes and insight into the evolutionary processes that led to the current associations of bats and their helminth parasites. Although a few studies on Malagasy bat ectoparasites [38] and endoparasites have been published [30, 32], information regarding parasites circulating in the island’s bat fauna is incomplete. Some insights have been presented on ectoparasites and viruses of Malagasy bats [15, 38] and different ongoing work on viruses, bacteria, and haemosporidian parasites of the same individual bats used in this study will provide a broad-scale understanding of their associated pathogens.

### Evidence of filarial infection in Malagasy bats

Malagasy bats are subject to filarial infection based on molecular detection carried out on 947 individuals, with an average infection rate of 6.8%. When these data are combined with adult filaria collected from hosts, the infection rate rises to 8.8%. This rate is similar to that found in a previous study [32] employing microscopic screening of thin blood smears, in which 7% of the individual tested bats (n = 414), representing 14 species, were found to be positive. Additionally, these authors reported the presence of blood microfilaria in a single individual of *Myotis goudoti* and in 30 individuals of *Miniopterus* “*manavi*”. Subsequently, “*M*. *manavi*” has been shown to be paraphyletic and now comprises at least six different species [33, 53, 54].

On Madagascar, different lineages of *Litomosa*, *Litomosoides-*related filaria, and a third distinct group of filarial nematodes appear to be largely host-family specific and associated with Miniopteridae, Vespertilionidae, and Vespertilionidae/Miniopteridae/Molossidae, respectively. Within *Miniopterus*, which was the most infected genus within our sample, rates were variable (number of positives/sample size): 5.2% (6/116) in *M*. *griveaudi*, 33.7% (30/89) in *M*. *mahafaliensis*, and 85% (6/7) in *M*. *majori*. Infection rates approaching 50% have been reported from South African *M*. *natalensis* [16, 24]; hence, *Litomosa* prevalence in certain Afro-Malagasy *Miniopterus* spp. appears to be high. Although any assumption regarding the evolutionary significance of such high infection prevalence is speculative, one may suppose it could confer to the infected animal some biological advantage, as recently shown in an experimental model of chronic infection of mice by *Litomosoides sigmodontis*: the filarial infection actually protected animals against the deleterious effects of acute *Escherichia coli* infection, improved bacterial clearance, and reduced the concentration of pro-inflammatory cytokines [55].

Our data also revealed that within Miniopteridae, males have a statistically significantly higher probability of being infected than females. Sex-biased parasitism is usually attributed to either ecological or physiological causes [56], the former associated with aspects of social behavior and the latter related to hormonal differences between sexes. We can best attribute this skewed sex ratio in Malagasy *Miniopterus* infection prevalence to their ecology, as field surveys revealed sexual segregation at roosting sites. For example, males dominated cave day-roost sites at Ambohitantely in the Central Highlands, where populations were composed of *M*. *manavi* and closely related forms, and at Andranomilitra Cave near Ihosy with *M*. *mahafaliensis*. In contrast, *M*. *sororculus* specimens sampled in Bekapity Cave in the Isalo region were all reproductively active females. At these localities and others, we found little evidence that both sexes share the same roosting site, although it is possible that they occur in different positions within a cave, such as bachelor colonies near the entrance and maternity colonies deeper within the system.

Beside the aforementioned cases, little quantitative data exist for sexual segregation of colonies on Madagascar. In southern Europe there is notable sexual division of day roost sites in *M*. *schreibersii* [57]. During the reproductive season, females form maternity colonies, which can also include yearlings of both sexes. In addition, female *M*. *schreibersii* are known to be philopatric [57, 58]. Such spatial separation between the sexes would provide differential exposure to arthropod vectors responsible for the transmission of filarial nematodes, specifically the Diptera families Culicidae, Psychodidae, and Ceratopogonidae [59]. On Madagascar, several dipteran families are known to show reduced occurrence and abundance across the gradient from areas just outside of caves, to the entrance twilight zones, and to dark interior sections [60]. Additional investigations are needed to further assess the relationship between sexual segregation of roosting sites and arthropod filaria vectors.

We detected sequences that are phylogenetically related to the genus *Litomosoides* in two specimens of *Pipistrellus* cf. *hesperidus*. Small bats within this family on Madagascar are difficult to differentiate based on external and cranio-dental characters [37]. Members of the genus *Litomosoides*, which are closely related to *Litomosa* [27, 61], are known to parasitize different Neotropical mammal groups, including rodents, marsupials, and bats [25, 27, 62–64]. In parallel with previous studies [22, 24], our analysis placed *Litomosa westi* within the *Litomosoides* group, rendering *Litomosa* paraphyletic [22, 24]. We did not recover adult filaria from the two positive *Pipistrellus* bats but only microfilaria from one specimen. Although our phylogenetic analysis embeds these two Malagasy sequences within the *Litomosoides* cluster, which was previously unknown from Madagascar (or anywhere in the Old World), adult filaria are needed to diagnose the generic placement of this nematode based on morphological and phylogenetic analyses.

### Filarial association within the *Miniopterus* species complex

We focused our analyses on filarial nematodes infecting *Miniopterus* spp., as these were the most prevalent and diverse host genus within our sample and, importantly, widespread across the island. Species within the family Miniopteridae can be divided into three groups based on body size: 1) large, composed of *M*. *gleni* and *M*. *griffithsi*, which are allopatric sister species [65]; 2) medium, including *M*. *majori* and *M*. *sororculus*, which tend to occur in the Central Highlands and have been found roosting in the same caves [66]; and 3) small, including *M*. *aelleni*, *M*. *brachytragos*, *M*. *griveaudi*, *M*. *mahafaliensis*, *M*. *manavi* sensu stricto, and *M*. *petersoni* that are all endemic to the Malagasy region (Madagascar and the Comoros Archipelago) [2, 33, 53]. In many cases, these different taxa can be found roosting in the same cave systems (Table 3).

**Table 3:** Syntopic associations (inter-species physical contact within roost-sites) of Malagasy *Miniopterus* spp.

	*M*. *aelleni*	*M*. *gleni*	*M*. *griffithsi*	*M*. *griveaudi*	*M*. *mahafaliensis*	*M*. *majori*	*M*. *manavi*	*M*. *sororculus*	Syntopic associations
*M*. *aelleni*	-								
*M*. *gleni*	Yes	-							1
*M*. *griffithsi*	No	No	-						0
*M*. *griveaudi*	Yes	Yes	No	-					2
*M*. *mahafaliensis*	No	Yes	Yes	No	-				2
*M*. *majori*	No	No	No	No	No	-			0
*M*. *manavi*	No	No	No	No	No	Yes	-		1
*M*. *sororculus*	No	Yes	No	No	Yes	Yes	No	-	3
Syntopic associations	2	3	1	0	3	2	1	3	

As presented in Fig 2, *Litomosa* clade 1 infected bats belonging to all three *Miniopterus* size classes, although with differing infection rates (highest in *M*. *majori*). For example, clade 1 was found in *M*. *gleni* from the northwest (Ankarana), *M*. *manavi* from the western Central Highlands (Ambohitantely), and *M*. *majori* and *M*. *sororculus* in the eastern Central Highlands (Fandanana). These latter two localities are separated from Ankarana by about 600 km and 820 km direct distance, respectively. *Litomosa* clade 2 infected *M*. *mahafaliensis*, *M*. *griffithsi*, *M*. *sororculus*, and *M*. *griveaudi*. *Miniopterus mahafaliensis* was the most heavily infected, being confirmed at seven of the nine sampled sites with nearly 33% of the samples positive by PCR (18 of the 30 PCR positive samples had adult filaria). *Miniopterus mahafaliensis* is known to occur within the same caves with *M*. *sororculus* or *M*. *griffithsi*, and it is likely that the filarial vectors are not host species specific.

### *Litomosa* species distribution and biogeography

In Fig 4, we present the known geographic distribution of different filarial *Litomosa* spp. on Madagascar overlaid on the island’s bioclimatic zones. Filarial nematodes infecting *Miniopterus* show geographic segregation in their distribution, which is also associated with different bioclimatic zones. *Litomosa* clade 1 occurred mainly in the Central Highlands (subhumid) and in the north (transitional subhumid-dry). *Litomosa* clade 2 was found mostly infecting *M*. *mahafaliensis* along the southwestern edge of the Central Highlands in the Isalo region (transitional subhumid-subarid), as well as in the central west (dry) to the extreme southwest (subarid). Among the 52 sampled sites, the Isalo Massif was the only one where both *Litomosa* clades 1 and 2 were found to co-occur and in hosts obtained at cave sites separated by 19 km direct distance.

The observed geographic patterns of *Litomosa* diversity may be associated with their host distribution or different factors such as altitude or bioclimatic conditions. Five species of *Miniopterus* (*M*. *griveaudi*, *M*. *gleni*, *M*. *majori*, *M*. *manavi*, and *M*. *sororculus*) were found infected with *Litomosa* clade 1. Some of these species live in syntopy and have been shown to share taxa of parasitic Diptera of the family Nycteribiidae [38]. These bats are probably exposed to vectors occurring in the same cave systems responsible for transmission of filarial nematodes. Such a multispecies system helps to insure the completion of the life cycle and the maintenance of the filarial parasites within the environment. In the case of *Litomosa* clade 2, the most common hosts were *M*. *mahafaliensis* and, to a lesser extent, *M*. *griffithsi*, *M*. *sororculus*, and *M*. *griveaudi*; in different combinations, these bat species are known to occur in sympatry (Table 3).

We provide herein evidence for a largely consistent geographic separation of the three clades of *Litomosa* occurring on Madagascar, which can, in part, be explained by environmental factors and are presumably correlated with filaria vectors. With regards to the life cycle of these parasites, filarial infection is characterized by the injection of third instar larvae via a blood-sucking arthropod vector [20]. No precise information is available regarding the full life cycle of *Litomosa* spp., and their invertebrate vectors are currently unknown. Junker *et al*. [24] proposed mites of the family Macronyssidae as possible vectors of larval *Litomosoides*, a filarial genus closely related to *Litomosa* spp. [25]. Future work should focus on the molecular screening of ectoparasites (bat flies, fleas, mites, and ticks) collected at sites where bats test positive for filarial nematodes, as well as blood-sucking Diptera. Furthermore, detailed studies combining morphological and molecular characters of filaria are needed to elucidate the systematic relationships of different clades and genera that are poorly known and better understand the biological cycle of these bat-infesting nematodes.

## Supporting Information

S1 Dataset Nexus alignment of the filarial COI sequences included in the present study. (NEX)

S1 Table Description of the different sites sampled across Madagascar. (DOC)

S2 Table Details of COI sequences of filarial nematodes included in the present study: isolates, marker, GenBank accession numbers, host, museum numbers, and origin. FMNH = Field Museum of Natural History, UADBA = Université d’Antananarivo, Département de Biologie Animale.
(DOC)

S3 Table Details of Cyt *b* sequences of *Miniopterus* used for Parafit analysis: marker, museum numbers, GenBank accession numbers, and origin. FMNH = Field Museum of Natural History, UADBA = Université d’Antananarivo, Département de Biologie Animale.
(DOC)
