# Supplementary material for: Diversity, Host Specialization, and Geographic Structure of Filarial Nematodes Infecting Malagasy Bats
Source: PLoS One. 2016 Jan 11;11(1):e0145709. doi: 10.1371/journal.pone.0145709 (PMC4709050; doi:10.1371/journal.pone.0145709)
Supplement: S1 Table — (DOC) [file pone.0145709.s002.doc]

S1 Table. Description of different sites sampled across Madagascar

| **Site** | **Province** | **Locality description** | **Latitude (°S)** | **Longitude (°E)** |
| --- | --- | --- | --- | --- |
| 1 | Antananarivo | Réserve Spéciale d’Ambohitantely, Grotte des Chauves-souris | -18.181167 | 47.28930 |
| 2 | Antananarivo | Réserve Spéciale d’Ambohitantely, début sentier touristique | -18.181167 | 47.28930 |
| 3 | Antananarivo | Sous préfecture d’Anjozorobe, Forêt d’Antsahabe, Andohasahabe | -18.410033 | 47.93678 |
| 4 | Antananarivo | Ambohibeloma, 3.2 km W d’Anjozorobe | -18.408820 | 47.85168 |
| 5 | Antsiranana | Parc National d’Ankarana, Ambahibe Cave, 2 km W Mahamasina | -12.967667 | 49.12052 |
| 6 | Antsiranana | Parc National d’Ankarana, Grotte des Chauves-souris, 3 km NW Mahamasina | -12.956317 | 49.11808 |
| 7 | Antsiranana | Parc National d’Ankarana, Grotte d’Andrafiabe, 3.3 ESE d’Andrafiabe | -12.931667 | 49.06000 |
| 8 | Antsiranana | Parc National d’Ankarana, 2.6 km E Andrafiabe, in forest near Andrafiabe Cave | -12.931667 | 49.05667 |
| 9 | Antsiranana | Parc National d’Ankarana, Grotte du troisième Canyon, along Andokotokana River | -12.914170 | 49.05500 |
| 10 | Antsiranana | Parc National d’Ankarana, 2.2 km ESE Amboandriky, Grotte d’Ambatoharanana | -12.988300 | 49.02170 |
| 11 | Fianarantsoa | Commune rurale d’Ankily, west of Ihosy off RN 7 | -22.385000 | 46.09556 |
| 12 | Fianarantsoa | Grotte d’Andranomilitra, west of Ihosy off RN 7 | -22.385240 | 46.05601 |
| 13 | Fianarantsoa | Grotte de Fandanana, 4.1 km NE de Fandriana | -20.182983 | 47.38552 |
| 14 | Fianarantsoa | Parc National d’Isalo, 3.8 km NW de Ranohira, along Namaza River | -22.540000 | 45.38000 |
| 15 | Fianarantsoa | Parc National d’Isalo, Grotte de Bekapity | -22.633336 | 45.21808 |
| 16 | Fianarantsoa | Edge of Parc National of Isalo, 7.8 km N Ranohira, along Menamaty River | -22.548000 | 45.39900 |
| 17 | Fianarantsoa | Parc National de l’Isalo, Ambinanindranohira-bas, Andranomboalavo | -22.485800 | 45.38680 |
| 18 | Fianarantsoa | Zazafotsy | -22.207080 | 46.36339 |
| 19 | Fianarantsoa | Ihosy, Bureau du chef de la Région, | -22.403360 | 46.12887 |
| 20 | Fianarantsoa | Vohiposa, CSB II | -20.995980 | 47.16156 |
| 21 | Mahajanga | Ambovondramanesy village near Berivotra, along RN 4 | -15.900000 | 46.58330 |
| 22 | Mahajanga | Mahajanga, Petite Plage | -15.667970 | 46.32205 |
| 23 | Mahajanga | Grotte d’Anjohikely (south entrance), 1.5 km NE d’Antanamarina | -15.560883 | 46.87420 |
| 24 | Mahajanga | Grotte d’Anjohibe, 3.7 km NE d’Antanamarina | -15.538150 | 46.88598 |
| 25 | Mahajanga | Grotte d’Anjohikely 2, 1.6 km NE d’Antanamarina | -15.558900 | 46.87750 |
| 26 | Mahajanga | Cascade d’Antanamarina | -15.576183 | 46.86928 |
| 27 | Mahajanga | Grotte de Beenta, 2 km W de Mitsinjo | -15.442510 | 46.90207 |
| 28 | Mahajanga | Parc National de Bemaraha, Anjohikinakina 15.5 km N de Bekopaka | -19.009900 | 44.76770 |
| 29 | Mahajanga | Limit of Parc National de Bemaraha, Ankapoka | -19.04490 | 44.77350 |
| 30 | Toamasina | Andasibe, CEG | -18.92016 | 48.41768 |
| 31 | Toamasina | Outskirts of Andasibe, Mangarivotra, Ambany Atsinanana | -18.91984 | 48.42044 |
| 32 | Toliara | Grotte de Sarodrano (sea cave) | -23.53000 | 43.73000 |
| 33 | Toliara | Grotte de Makis (Mikea), near Hotel la Mangrove on Toliara-St. Augustin Road | -23.47211 | 43.77069 |
| 34 | Toliara | Grotte de Bekoaky, 9.4 km SSE d’Ankililaoka | -22.77300 | 43.72267 |
| 35 | Toliara | Grotte de Tanambao (Bishiko), 0.75 km E de St. Augustin | -23.54888 | 43.76740 |
| 36 | Toliara | Grotte d'Ambanilia, 3.7 km SSE de Sarodrano | -23.53995 | 43.74605 |
| 37 | Toliara | St. Augustin, in lycée building | -23.54930 | 43.75720 |
| 38 | Toliara | Parc National de Tsimanampetsotsa, Grotte d’Andranoilovy | -24.05000 | 43.75000 |
| 39 | Toliara | Grotte d’Androimpano, 4.2 km NE d’Itampolo (village), on old road to Ejeda | -24.48353 | 43.96328 |
| 40 | Toliara | Itampolo (village) | -24.68431 | 43.94583 |
| 41 | Toliara | Grotte de Vintane (Vintany), 4.1 km SE d’Itampolo | -24.70236 | 43.96372 |
| 42 | Toliara | Betioky Sud, New Lutheran Church | -23.71986 | 44.38350 |
| 43 | Toliara | Sakaraha, Direction des Eaux et forêts, Bureau chef de cantonnement | -22.90946 | 44.52279 |
| 44 | Toliara | Sakaraha, near Direction des Eaux et forêts complex at edge of town | -22.90900 | 44.52300 |
| 45 | Toliara | Marofandilia (village), Ecole primaire | -20.06743 | 44.65800 |
| 46 | Toliara | Kirindy (village) | -20.06332 | 44.59679 |
| 47 | Toliara | Antanandava, Eglise FLM, 5.8 km NE de Beroboka Sud | -19.92973 | 44.60594 |
| 48 | Toliara | Tsimafana, CEG de Tsimafana | -19.72350 | 44.58432 |
| 49 | Toliara | 0.8 km N de Kirindy (village) | -20.06222 | 44.60126 |
| 50 | Toliara | Marofototra, Tsarafototra FLM | -20.30244 | 44.39807 |
| 51 | Toliara | Mahabo, EPP de Mahabo | -20.37770 | 44.66072 |
| 52 | Toliara | Belo Tsiribihina, Central Hospital | -19.70040 | 44.54750 |
