# Supplementary material for: Diversity, Host Specialization, and Geographic Structure of Filarial Nematodes Infecting Malagasy Bats
Source: PLoS One. 2016 Jan 11;11(1):e0145709. doi: 10.1371/journal.pone.0145709 (PMC4709050; doi:10.1371/journal.pone.0145709)
Supplement: S2 Table — FMNH = Field Museum of Natural History, UADBA = Université d’Antananarivo, Département de Biologie Animale. (DOC) [file pone.0145709.s003.doc]

S2 Table. Details of COI sequences of filarial nematodes included in the present study: isolates, marker, GenBank accession numbers, host, museum numbers, and origin. FMNH = Field Museum of Natural History, UADBA = Université d’Antananarivo, Département de Biologie Animale.

| **Species** | **Isolates** | **Marker** | **GenBank n°** | **Host** | **Host Museum number** | **Origin** |
| --- | --- | --- | --- | --- | --- | --- |
| *Litomosa* Clade 2 | 36MG | COI | KP728027 | *Miniopterus mahafaliensis* | FMNH 217957 | Madagascar |
| *Litomosa* Clade 2 | 37MG | COI | KP728028 | *Miniopterus mahafaliensis* | FMNH 217958 | Madagascar |
| *Litomosa* Clade 2 | 72MG | COI | KP728031 | *Miniopterus mahafaliensis* | UADBA 32352 | Madagascar |
| *Litomosa* Clade 2 | 73MG | COI | KP728032 | *Miniopterus mahafaliensis* | FMNH 217971 | Madagascar |
| *Litomosa* Clade 2 | 74MG | COI | KP728033 | *Miniopterus mahafaliensis* | UADBA 32353 | Madagascar |
| *Litomosa* Clade 2 | 75MG | COI | KP728034 | *Miniopterus mahafaliensis* | UADBA 32354 | Madagascar |
| *Litomosa* Clade 2 | 76MG | COI | KP728035 | *Miniopterus mahafaliensis* | UADBA 32355 | Madagascar |
| *Litomosa* Clade 2 | 101MG | COI | KP728036 | *Miniopterus mahafaliensis* | UADBA 32366 | Madagascar |
| *Litomosa* Clade 2 | 104MG | COI | KP728037 | *Miniopterus mahafaliensis* | UADBA 32369 | Madagascar |
| *Litomosa* Clade 2 | 116MG | COI | KP728038 | *Miniopterus sororculus* | FMNH 217986 | Madagascar |
| *Litomosa* Clade 2 | 117MG | COI | KP728039 | *Miniopterus sororculus* | FMNH 217987 | Madagascar |
| *Litomosa* Clade 2 | 118MG | COI | KP728040 | *Miniopterus mahafaliensis* | FMNH 217988 | Madagascar |
| *Litomosa* Clade 2 | 119MG | COI | KP728041 | *Miniopterus mahafaliensis* | FMNH 217989 | Madagascar |
| *Litomosa* Clade 2 | 50MG | COI | KP728029 | *Miniopterus mahafaliensis* | UADBA 32371 | Madagascar |
| *Litomosa* Clade 2 | 121MG | COI | KP728042 | *Miniopterus mahafaliensis* | FMNH 217991 | Madagascar |
| *Litomosa* Clade 2 | 122MG | COI | KP728043 | *Miniopterus mahafaliensis* | FMNH 217992 | Madagascar |
| *Litomosa* Clade 2 | 123MG | COI | KP728044 | *Miniopterus mahafaliensis* | FMNH 217993 | Madagascar |
| *Litomosa* Clade 1 | 723MG | COI | KP728073 | *Miniopterus sororculus* | UADBA 50172 | Madagascar |
| *Litomosa* Clade 1 | 724MG | COI | KP728074 | *Miniopterus sororculus* | UADBA 50173 | Madagascar |
| *Litomosa* Clade 1 | 725MG | COI | KP728075 | *Miniopterus majori* | UADBA 50136 | Madagascar |
| *Litomosa* Clade 1 | 726MG | COI | KP728076 | *Miniopterus majori* | UADBA 50137 | Madagascar |
| *Litomosa* Clade 1 | 728MG | COI | KP728077 | *Miniopterus majori* | FMNH 218008 | Madagascar |
| *Litomosa* Clade 1 | 729MG | COI | KP728078 | *Miniopterus majori* | FMNH 218009 | Madagascar |
| *Litomosa* Clade 1 | 730MG | COI | KP728079 | *Miniopterus majori* | FMNH 218010 | Madagascar |
| *Litomosa* Clade 1 | 737MG | COI | KP728080 | *Miniopterus majori* | FMNH 218011 | Madagascar |
| *Litomosa* Clade 2 | 741MG | COI | KP728081 | *Miniopterus mahafaliensis* | UADBA 50162 | Madagascar |
| *Litomosa* Clade 2 | 742MG | COI | KP728082 | *Miniopterus mahafaliensis* | FMNH 218040 | Madagascar |
| *Litomosa* Clade 2 | 757MG | COI | KP728083 | *Miniopterus mahafaliensis* | FMNH 218047 | Madagascar |
| *Litomosa* Clade 1 | 772MG | COI | KP728085 | *Miniopterus sororculus* | UADBA 50344 | Madagascar |
| *Litomosa* Clade 2 | 150MG | COI | KP728045 | *Miniopterus mahafaliensis* | UADBA 50184 | Madagascar |
| *Litomosa* Clade 2 | 175MG | COI | KP728046 | *Miniopterus griffithsi* | FMNH 218063 | Madagascar |
| *Litomosa* Clade 2 | 189MG | COI | KP728047 | *Miniopterus mahafaliensis* | UADBA 50217 | Madagascar |
| *Litomosa* Clade 2 | 190MG | COI | KP728048 | *Miniopterus mahafaliensis* | UADBA 50218 | Madagascar |
| *Litomosa* Clade 2 | 191MG | COI | KP728049 | *Miniopterus mahafaliensis* | UADBA 50219 | Madagascar |
| *Litomosa* Clade 2 | 193MG | COI | KP728050 | *Miniopterus mahafaliensis* | UADBA 50221 | Madagascar |
| *Litomosa* Clade 2 | 194MG | COI | KP728051 | *Miniopterus mahafaliensis* | FMNH 218073 | Madagascar |
| *Litomosa* Clade 2 | 195MG | COI | KP728052 | *Miniopterus mahafaliensis* | FMNH 218074 | Madagascar |
| *Litomosa* Clade 2 | 196MG | COI | KP728053 | *Miniopterus mahafaliensis* | UADBA 50222 | Madagascar |
| *Litomosa* Clade 2 | 199MG | COI | KP728054 | *Miniopterus mahafaliensis* | FMNH 218076 | Madagascar |
| *Litomosa* Clade 2 | 201MG | COI | KP728055 | *Miniopterus mahafaliensis* | FMNH 218078 | Madagascar |
| *Litomosa* Clade 2 | 202MG | COI | KP728056 | *Miniopterus mahafaliensis* | FMNH 218079 | Madagascar |
| *Litomosa* Clade 3 | 504MG | COI | KP728059 | *Miniopterus griveaudi* | FMNH 221340 | Madagascar |
| *Litomosa* Clade 1 | 543MG | COI | KP728063 | *Miniopterus griveaudi* | UADBA 33026 | Madagascar |
| *Litomosa* Clade 1 | 585MG | COI | KP728064 | *Miniopterus gleni* | UABBA 33029 | Madagascar |
| *Litomosa* Clade 1 | 589MG | COI | KP728065 | *Miniopterus gleni* | FMNH 221331 | Madagascar |
| *Litomosa* Clade 1 | 592MG | COI | KP728066 | *Miniopterus gleni* | FMNH 221334 | Madagascar |
| *Litomosa* Clade 1 | 608MG | COI | KP728067 | *Miniopterus manavi* | UADBA 33035 | Madagascar |
| *Litomosa* Clade 1 | 613MG | COI | KP728068 | *Miniopterus manavi* | UADBA 33040 | Madagascar |
| *Litomosa* Clade 1 | 615MG | COI | KP728069 | *Miniopterus manavi* | FMNH 221421 | Madagascar |
| *Litomosa* Clade 1 | 972MG | COI | KP728089 | *Miniopterus manavi* | FMNH 221422 | Madagascar |
| *Litomosa* Clade 1 | 53MG | COI | KP728030 | *Miniopterus manavi* | FMNH 221429 | Madagascar |
| *Litomosa* Clade 3 | 649MG | COI | KP728070 | *Miniopterus aelleni* | FMNH 221440 | Madagascar |
| *Litomosa* Clade 2 | 655MG | COI | KP728071 | *Miniopterus griveaudi* | UADBA 33678 | Madagascar |
| *Litomosa* Clade 3 | 656MG | COI | KP728072 | *Miniopterus aelleni* | FMNH 221444 | Madagascar |
| *Litomosa* Clade 2 | 834MG | COI | KP728086 | *Miniopterus mahafaliensis* | FMNH 222720 | Madagascar |
| Unnamed filarioid | 506MG | COI | KP728060 | *Miniopterus griveaudi* | FMNH 221343 | Madagascar |
| Unnamed filarioid | 516MG | COI | KP728061 | *Miniopterus griveaudi* | UADBA 33012 | Madagascar |
| Unnamed filarioid | 518MG | COI | KP728062 | *Miniopterus griveaudi* | UADBA 33014 | Madagascar |
| Unnamed filarioid | 760MG | COI | KP728084 | *Myotis goudoti* | UADBA 50348 | Madagascar |
| Unnamed filarioid | 861MG | COI | KP728087 | *Neoromicia matroka* | FMNH 222725 | Madagascar |
| Unnamed filarioid | 961MG | COI | KP728088 | *Otomops madagascariensis* | UADBA 32925 | Madagascar |
| *Litomosa* Clade 1 | Fil4 | COI | KP728093 | *Miniopterus manavi* | UADBA 50504 | Madagascar |
| *Litomosa* Clade 1 | Fil5 | COI | KP728094 | *Miniopterus manavi* | FMNH 225988 | Madagascar |
| *Litomosa* Clade 2 | Fil1 | COI | KP728090 | *Miniopterus mahafaliensis* | UADBA 50182 | Madagascar |
| *Litomosa* Clade 2 | Fil2 | COI | KP728091 | *Miniopterus mahafaliensis* | UADBA 50216 | Madagascar |
| *Litomosa* Clade 2 | Fil3 | COI | KP728092 | *Miniopterus mahafaliensis* | FMNH 218078 | Madagascar |
| *Litomosoides* sp. | 467MG | COI | KP728057 | *Pipistrellus* cf. *hesperidus* | UADBA 32936 | Madagascar |
| *Litomosoides* sp. | 468MG | COI | KP728058 | *Pipistrellus* cf. *hesperidus* | UADBA 32937 | Madagascar |
| *Wuchereria bancrofti* |  | COI | AJ271612 | *Homo sapiens* |  | Sri Lanka |
| *Wuchereria bancrofti* |  | COI | AM749235 | *Homo sapiens* |  | Italy |
| *Litomosoides brasiliensis* |  | COI | AJ544867 | *Carollia persillata* |  | Venezuela |
| *Litomosoides* sp. |  | COI | FR719324 | *Nectomys palmipes* |  | Venezuela |
| *Litomosa westi* |  | COI | AJ544871 | *Geomys bursarius* |  | USA |
| *Setaria labiatopapillosa* |  | COI | AJ544872 | *Bos taurus* |  | Italy |
| *Loa loa* |  | COI | AJ544875 | *Homo sapiens* |  | Cameroon |
| *Brugia pahangi* |  | COI | DQ977746 |  |  | Malaysia |
| *Litomosoides scotti* |  | COI | EF661995 |  |  |  |
| *Dirofilaria immitis* | GEN3 | COI | AM749228 | *Canis lupus* |  | Italy |
| *Dirofilaria repens* | CATM | COI | AM749232 | *Felis catus* |  | Italy |
| *Litomosoides yutajensis* | 39 YU | COI | AM749280 | *Pteronotus parnelii* |  | Venezuela |
| *Litomosoides sigmodontis* | M1L | COI | AM749286 | *Sigmodon hispidus* |  | Paris |
| *Litomosa chiropterorum* |  | COI | FM209527 | *Miniopterus natalensis* |  | South Africa |
| *Litomosa chiropterorum* |  | COI | FM209529 | *Miniopterus natalensis* |  | South Africa |
| *Litomosa chiropterorum* |  | COI | FM209530 | *Miniopterus natalensis* |  | South Africa |
| *Litomosa chiropterorum* |  | COI | FM209531 | *Miniopterus natalensis* |  | South Africa |
| *Litomosa chiropterorum* |  | COI | FM209532 | *Miniopterus natalensis* |  | South Africa |
| *Litomosa chiropterorum* |  | COI | FM209533 | *Miniopterus natalensis* |  | South Africa |
| *Litomosa chiropterorum* |  | COI | FM209534 | *Miniopterus natalensis* |  | South Africa |
| *Litomosa chiropterorum* |  | COI | FM209535 | *Miniopterus natalensis* |  | South Africa |
| *Spirocerca lupi* | Isolate n. 5 | COI | EF394602 | *Canis* sp. |  | Italy |
| *Spirocerca lupi* | Isolate n. 6 | COI | EF394603 | *Canis* sp. |  | Iran |
| *Spirocerca lupi* | Isolate n. 8 | COI | EF394604 | *Canis* sp. |  | Israel |
| *Spirocerca lupi* | Isolate n .9 | COI | EF394605 | *Canis* sp. |  | Austria |
| *Thelazia callipaeda* |  | COI | AM042553 |  |  | China |
| *Thelazia callipaeda* |  | COI | AM042554 |  |  | China |
| *Thelazia callipaeda* |  | COI | AM042555 |  |  | China |
| *Thelazia callipaeda* |  | COI | AM042556 |  |  | China |
| *Thelazia lacrymalis* |  | COI | AJ271619 |  |  |  |
