# Supplementary material for: Diversity, Host Specialization, and Geographic Structure of Filarial Nematodes Infecting Malagasy Bats
Source: PLoS One. 2016 Jan 11;11(1):e0145709. doi: 10.1371/journal.pone.0145709 (PMC4709050; doi:10.1371/journal.pone.0145709)
Supplement: S3 Table — FMNH = Field Museum of Natural History, UADBA = Université d’Antananarivo, Département de Biologie Animale. (DOC) [file pone.0145709.s004.doc]

S3 Table. Details of Cyt *b* sequences of *Miniopterus* used for Parafit analysis: marker, museum numbers, GenBank accession numbers, and origin. FMNH = Field Museum of Natural History, UADBA = Université d’Antananarivo, Département de Biologie Animale.

| **Bat species** | **Marker** | **Museum number** | **GenBank number** | **Origin** |
| --- | --- | --- | --- | --- |
| *M. manavi* | *Cyt b* | UADBA 43171 | HQ619934 | Madagascar |
| *M. aelleni* | *Cyt b* | FMNH-SMG 16646 | JF440225 | Madagascar |
| *M. gleni* | *Cyt b* | UADBA 43219 | JF440235 | Madagascar |
| *M. griffithsi* | *Cyt b* | UADBA 43229 | JF440240 | Madagascar |
| *M. griveaudi* | *Cyt b* | FMNH-SMG 16725 | JF440262 | Madagascar |
| *M. mahafaliensis* | *Cyt b* | FMNH 209279 | JF440268 | Madagascar |
| *M. majori* | *Cyt b* | UADBA 43254 | JF440276 | Madagascar |
| *M. sororculus* | *Cyt b* | UADBA 43202 | JF440284 | Madagascar |
| *M. natalensis* | *Cyt b* | ECJS 86/2008 | KF709541 | South Africa |
